# Eating Disorders in Adolescents and Young Adults: A Program Evaluation of a Canadian Eating Disorder Treatment Program

**DOI:** 10.7759/cureus.74478

**Published:** 2024-11-26

**Authors:** Caseita Dewar-Morgan, Pria Nippak, Housne Begum, Shannon Remers, Zahava Rosenberg-Yunger, Julien M Meyer, Alaina Nippak

**Affiliations:** 1 Health Services Management, Ted Rogers School of Management, Toronto Metropolitan University, Toronto, CAN; 2 Research, Quality, and Outcomes, Homewood Health Inc., Guelph, CAN

**Keywords:** adolescents and young adults, anorexia nervosa, anxiety, bulimia nervosa, depression, eating disorders, eating psychopathology, group cognitive behavioral therapy

## Abstract

Background: Current treatments for adolescents with eating disorders (ED) show limited effectiveness, emphasizing the need for enhanced therapeutic approaches. Cognitive behavioral therapy (CBT) has emerged as a potential alternative. A derivative of this approach, group cognitive behavioral therapy (G-CBT), has been shown to reduce treatment costs and increase treatment accessibility when compared to CBT. This program evaluation aimed to assess the effectiveness of G-CBT in adolescents and young adults diagnosed with anorexia nervosa (AN) or bulimia nervosa (BN) and experiencing comorbid anxiety and depression within a Canadian mental health facility. The specific objectives were to determine if participants increased their knowledge about eating normalization and coping strategies after participating in the eating disorder (ED) treatment program and examine if participants experienced changes in eating-disordered behavior, anxiety, and mental health.

Methods: We conducted a program evaluation using secondary data collected at admission and discharge from 44 adolescents and young adults (16-39 years) with AN or BN participating in the ED program at a Canadian health center. Outcome measures were eating psychopathology, depression, anxiety, and illness cognitions assessed using validated tools: Eating Disorder Examination Questionnaire (EDE-Q), Patient Health Questionnaire-9 (PHQ-9), General Anxiety Disorder-7 (GAD-7), and Illness Cognition Scale (ICS).

Results: The mean age of participants was 24.1 years (standard deviation (SD) = 5.8). All outcome measures showed statistically significant improvement from admission to discharge (p < 0.001). Specifically, subscales of the EDE-Q (eating concern, weight concern, shape concern, and restraint) and the global score indicated a significant reduction in ED behaviors (p < 0.001).

Conclusion: Findings suggest that G-CBT is effective in reducing eating-disordered behavior, depression, anxiety, and maladaptive illness cognitions in adolescents and young adults with EDs. These results underscore the potential of G-CBT to address both behavioral and psychological aspects of ED recovery, although further studies with control groups are warranted to confirm these findings.

## Introduction

Eating disorders (EDs) are psychiatric disorders described by a drastic disturbance in body weight as well as eating behavior. From 2000 to 2018, approximately 1.7 million Canadians were reported to have EDs, the majority of which were female adolescents and young adults [1]. Existing treatment options for adolescents and young adults with eating disorders (EDs) have shown limited effectiveness, underscoring an urgent need for innovative and more effective therapeutic approaches [2]. Cognitive behavioral therapy (CBT) has emerged as a potential alternative [3,4]. Group cognitive behavioral therapy (G-CBT), a structured adaptation of CBT, offers potential advantages by reducing treatment costs and enhancing accessibility in comparison to individual CBT [5].

According to the Diagnostic and Statistical Manual of Mental Disorders, Fifth Edition (DSM-5), EDs encompass a range of disorders, including anorexia nervosa (AN), bulimia nervosa (BN), binge eating disorder (BED), and others such as avoidant/restrictive food intake disorder (ARFID) and other specified feeding or eating disorder (OSFED) [6]. EDs lead to extensive physical and psychological health issues, often resulting in severe medical complications that compromise quality of life and, in some cases, become life-threatening [7]. Most medical complications result from malnutrition, while others result from the effects of purging and electrolyte imbalance [8]. For example, long-term electrolyte imbalances often result in cardiovascular diseases, which can lead to sudden death when combined with severe malnutrition [8]. Moreover, gastrointestinal diseases, another medical complication from EDs, can develop from purging behaviors such as self-induced vomiting and laxative abuse along with other medical complications such as endocrine and musculoskeletal complications.

In addition to physical health complications, EDs are often accompanied by comorbid mental health conditions. Martín et al. [9] and Robinson and Deane [10] have reported that anxiety and depression are highly comorbid with EDs. Moreover, AN is associated with the highest mortality rate of any mental illness, and one in five deaths is the result of suicide [11]. The National Initiative for Eating Disorders states that the second leading cause of mortality in individuals with AN in Canada is suicide, and suicide is attempted by 20% of people with AN and 25%-35% of those with BN [12].

Despite high mortality rates [8,12] associated with EDs, especially among adolescents and young adults, current treatments remain limited in effectiveness. This highlights the necessity for therapeutic strategies that specifically address both the behavioral and psychological impacts of EDs in this age group [2]. CBT has proven to be an effective treatment for older individuals with ED and is showing promising results in adolescent populations [13-16]. A derivative of this approach, group cognitive behavioral therapy (G-CBT) has been shown to reduce treatment costs and increase treatment accessibility when compared to CBT [5]. Moreover, it is suggested that G-CBT might be more effective due to its capacity to normalize experiences associated with EDs that may be considered shameful [17]. These benefits may encourage youth to seek treatment, and previous research suggests that G-CBT may achieve good results with adolescents facing other kinds of mental health challenges [18]. However, limited information is available on its use for adolescents and young adults diagnosed with AN or BN.

To address this gap, the current evaluation investigates the effectiveness of a G-CBT treatment program specifically designed for adolescents and young adults diagnosed with AN or BN at a major Canadian mental health and addiction facility. This addiction facility provides care that addresses the complex needs of patients and comorbid conditions delivering patient-centered treatment through an interdisciplinary team model. The ED program provides a strong community milieu and body-positive culture through coed group-based programming. Specifically, this program evaluation objectives were to (1) determine whether participants increased their knowledge about eating normalization and coping strategies after participating in the ED treatment program and (2) examine whether participants exhibited changes in eating-disordered behavior as well as anxiety and depression.

## Materials and methods

Study design

This program evaluation utilized retrospective secondary data gathered at both admission and discharge from participants at Homewood Health Inc., a prominent Canadian mental health and addiction facility. This evaluation involved comparing scores from four validated assessment tools (detailed below) to assess the program's effectiveness in alleviating psychological and behavioral challenges related to eating psychopathology.

Participants

**Figure 1:**
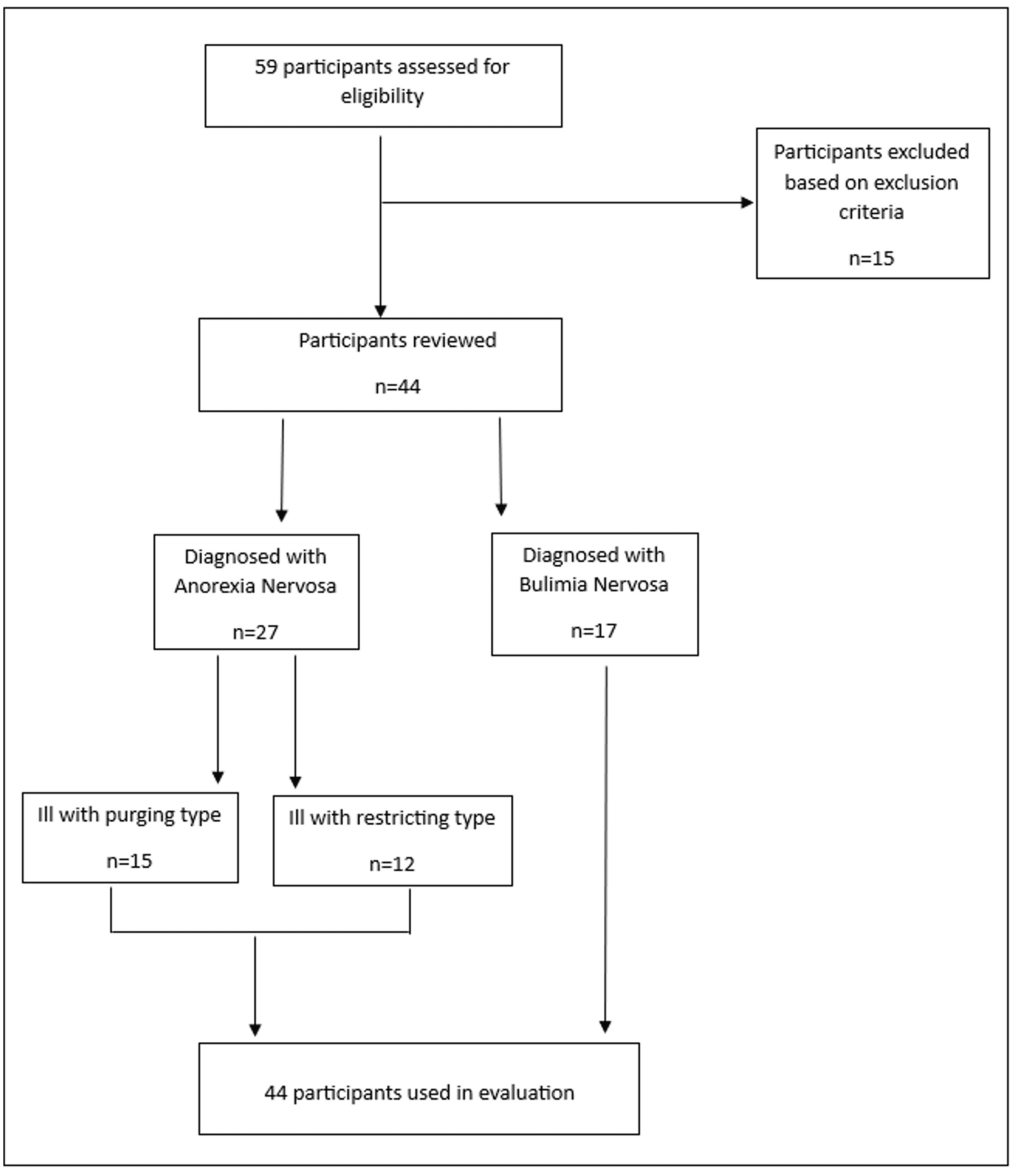
Participant flowchart n: number of participants

The participants in the program evaluation completed an eating disorder treatment program at Homewood Health Inc., one of the largest mental health and addiction facilities in Canada. Eligibility criteria included the following: (1) adolescents and young adults aged 16-39 years, (2) a diagnosis of anorexia nervosa (AN) or bulimia nervosa (BN) based on DSM-5 criteria, and (3) active participation in the ED program between January 2019 and February 2021 (Figure 1). This age group was selected because individuals in this demographic are at a higher risk of suicide. The National Initiative for Eating Disorders states that the AN mortality rate in females aged 15-24 is 12 times greater than all other combinations of causes of death [12], while Arcelus et al. show that the highest standardized mortality ratio of EDs occurs in the 15-30+ age group [19]. Participants who did not meet the inclusion criteria were excluded. Ethical approval for this evaluation was granted by the Toronto Metropolitan University Research Ethics Board (REB 2022-294).

Treatment program

Treatment was facilitated by a multidisciplinary team of clinicians, with dialectical behavioral therapy (DBT) and CBT laying the foundation of the program and skills and psychoeducation components. The G-CBT program was developed with group content that is evidence-informed by CBT and eating disorders by Fairburn et al. [20]. The program spans 16 weeks and includes programming that focuses on ED recovery but also allows room for focus on comorbid disorders and weight restoration, which can increase this timeframe. The intervention included weekly 90-minute sessions divided into psychoeducation and process groups. Psychoeducation sessions provided topic-specific information, while process groups facilitated interpersonal engagement among participants.

COVID-19 and the program

The health center followed public health recommendations, including masking, limiting groups, and physical distancing during the COVID-19 pandemic. The program remained open, and participants continued in the program during this time. The groups were offered both in person and virtually (when needed), which is contrary to pre-pandemic, where all meetings were in-person.

Measurement tools

The evaluation used validated outcome measurement tools [21-24] already implemented at Homewood Health Inc. to assess the psychological and behavioral aspects of EDs. No specialized training in psychometric assessments was required to administer these assessments.

Outcome measurement tools

Eating Disorder Examination Questionnaire (EDE-Q)

The Eating Disorder Examination Questionnaire (EDE-Q) is a widely used self-report measure of eating-disordered behaviors, adapted from the interview-based Eating Disorder Examination (EDE) [22]. It includes 33 items. Questions 1-12 and 19-28 are scored on a 7-point scale (0-6), and all questions (1-28) measure cognitive and behavioral characteristics of EDs that occurred within the previous 28 days. The severity of ED is determined by the total scores with the most severe being indicated by higher scores. The final five questions are concerned with weight, height, menstruation, and the contraceptive pill.

Patient Health Questionnaire-9 (PHQ-9)

PHQ-9 is a nine-item self-administered version of the depression portion of the Primary Care Evaluation of Mental Disorders that is used to measure mental disorders [23] and is based on occurrence within the previous two weeks. Scoring is based on a 4-point scale with scores ranging from 0 (not at all) to 3 (nearly every day). As a result, severity measures can range from 0 to 27.

General Anxiety Disorder-7 (GAD-7)

GAD-7 is a seven-item self-report that measures anxiety. Scoring is based on a 4-point scale with scores ranging from 0 (not at all) to 3 (nearly every day). As a result, severity measures can range from 0 to 21. GAD-7 scores of 5-9 represent mild, 10-14 are moderate, and ≥15 is severe [24].

Illness Cognition Scale (ICS)

ICS is a questionnaire that consists of 17 questions that measure attitudes and thoughts toward illness in relation to a reluctance to adapt to not being sick anymore [21]. It uses a Likert scale with responses of "strongly agree," "agree," "neither agree nor disagree," "disagree," and "strongly disagree" for scoring with question 12 being reverse scored.

Procedures

**Figure 2:**
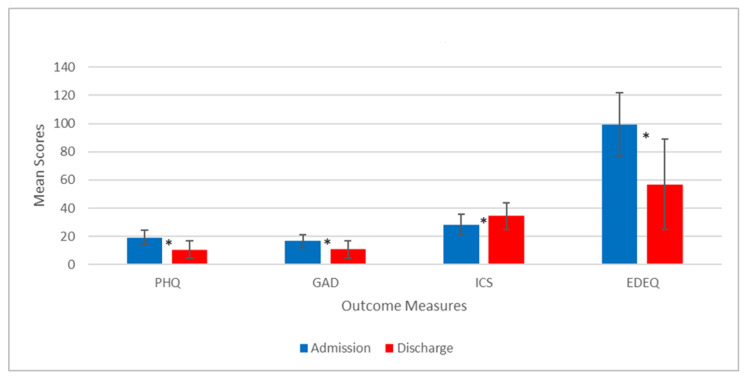
Mean scores at admission and discharge *p < 0.001 PHQ: Patient Health Questionnaire, GAD: General Anxiety Disorder, ICS: Illness Cognition Scale, EDE-Q: Eating Disorder Examination Questionnaire

Participants completed outcome measures at both admission and discharge, with scores recorded to assess changes over the course of the ED treatment program. These assessments were conducted by members of the interdisciplinary team, who had participants complete the paper-based measures at the start of the program and again upon discharge (Figure 2).

Missing data

**Figure 3:**
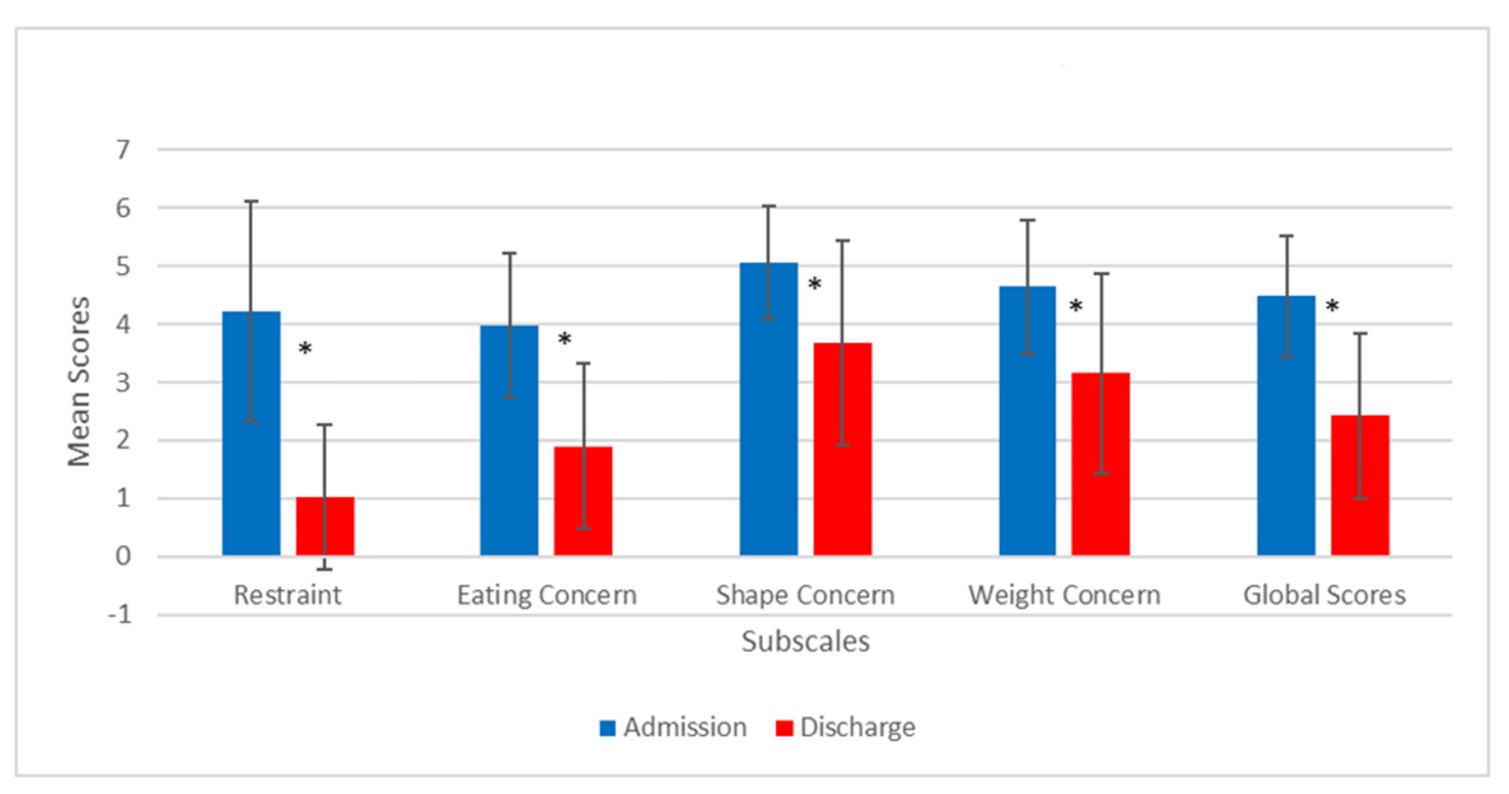
Mean scores for subscales at admission and discharge *p < 0.001

**Figure 4:**
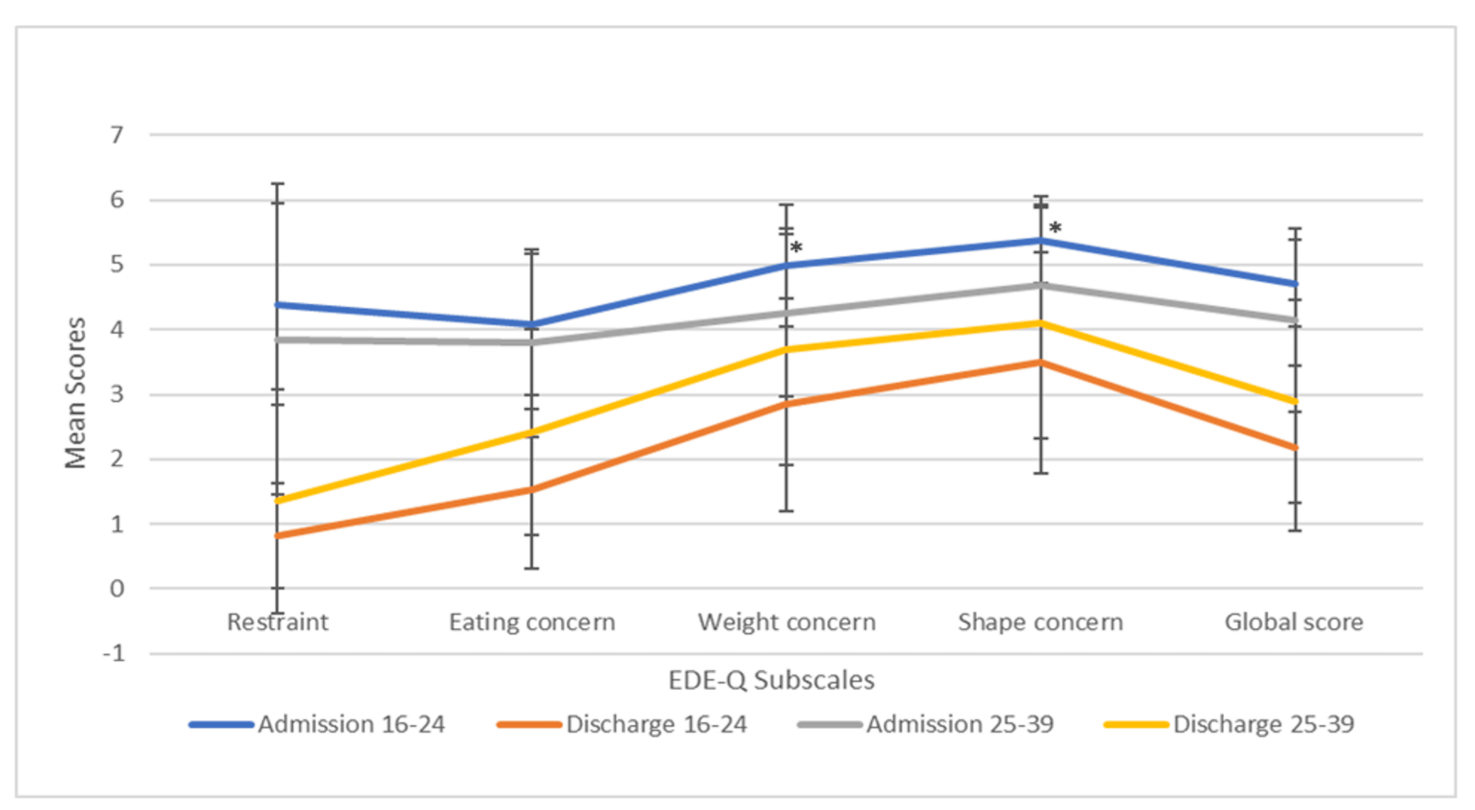
Mean scores for subscales by age group *p < 0.1 EDE-Q: Eating Disorder Examination Questionnaire

For the calculation of the EDE-Q subscales (restraint, eating concern, shape concern, and weight concern) and global scores, the methods recommended by Fairburn and Cooper were followed, that is, subscale scores were calculated when data for more than half of the relevant items were available, while the global score was calculated when scores on more than half (i.e., three or four) of the four subscales were available (Figure 3 and Figure 4) [15]. By following the method of Fairburn and Cooper, 30 participants' EDE-Q scores were included for subscales analysis.

Statistical analyses

Analyses included a comparison of assessment scores from admission to discharge, stratified by diagnostic groups. All analyses were performed using the statistical software SPSS (IBM Corp., Armonk, NY). Descriptive statistics were calculated, and means were presented with standard deviations (SDs) for demographic and baseline clinical characteristics. Overall scores were calculated for each tool for each participant for admission and discharge along with subscales and global scores for the EDE-Q scores. Data for the subscales are presented as mean scores on each subscale for individuals in each age group. Correlation analyses were performed to assess associations between demographic characteristics and different continuous variables. Paired t-tests were used to compare changes in scores between admissions and discharge. One-way analysis of variance (ANOVA) was used to test for differences in subscale scores across age groups, and post hoc tests were used to clarify the source of any effects significant at the p < 0.05 level.

## Results

Participant characteristics

Of the initial 59 participants, 44 (75%) met the eligibility criteria for inclusion in the evaluation. This sample included 15 participants with anorexia nervosa (AN) binge eating/purging type, 12 with AN restricting type (total AN = 27), and 17 with bulimia nervosa (BN).

The evaluation sample consisted entirely of female participants, with a mean age of 24.1 years (SD = 5.8). Most participants were single (86%), and their educational backgrounds varied, with 43% having some college or university experience, 20% holding a high school diploma, and 18% possessing a diploma or bachelor's degree. Table 1 presents the demographic characteristics of the participants.

**Table 1:** Participants' characteristics SD: standard deviation, n: number of participants

Characteristic	Participants (n = 44)
Gender (number (%))	
Female	44 (100)
Age in years	
Mean (SD)	24.1 (5.8)
Diagnosis (number (%))	
Anorexia nervosa, binge eating/purging type	15 (34)
Anorexia nervosa, restricting type	12 (27)
Bulimia nervosa	17 (39)
Marital status (number (%))	
Never married (single)	38 (86)
Married/common law	1 (2)
Partner/significant other	5 (11)
Education level (number (%))	
8 grade or less	1 (2)
9-11 grades	4 (9)
High school	9 (20)
Some college or university	19 (43)
Diploma or bachelor's degree	8 (18)
Graduate degree	3 (7)

Comparison of outcome scores at admission and discharge

Table 2 presents mean scores, standard deviations, and the minimum and maximum values for each tool, comparing admission and discharge scores. The minimum sum of scores for severity measures at admission for PHQ, GAD, ICS, and EDE-Q are 3, 1, 11, and 42, respectively, with a maximum of 27, 21, 40, and 130, respectively. At discharge, the minimum sum of scores for severity measures are 0, 0, 2, and 6, respectively, and the maximum are 25, 21, 53, and 132, respectively. All outcomes are significant (p < 0.001).

**Table 2:** Comparison of outcome scores at admission and discharge Data are shown as number and mean (±SD) unless otherwise stated. ^a^Significance of comparison according to a paired t-test ^b^Patient Health Questionnaire-9 ^c^General Anxiety Disorder-7 ^d^Illness Cognition Scale ^e^Eating Disorder Examination Questionnaire *n = 38, **n = 30 Min: minimum, Max: maximum, SD: standard deviation

	Admission	Min	Max	Discharge	Min	Max	p^a^
PHQ^b^	19.23 (±5.19)	3	27	10.59 (±6.27)	0	25	<0.001
GAD^c^	16.61 (±4.72)	1	21	10.80 (±6.14)	0	21	<0.001
ICS^d*^	28.24 (±7.33)	11	40	34.42 (±9.45)	2	53	<0.001
EDE-Q^e**^	99.40 (±22.35)	42	130	56.93 (±31.89)	6	132	<0.001

Relationship analyses between demographic characteristics and outcome scores

Correlation analyses assessed associations between demographic characteristics and outcome variables. No significant associations were observed between outcome improvements and demographic factors such as marital status, diagnosis, or education level (all p > 0.05). The results from the correlational analyses that assessed the associations between demographic characteristics and EDE-Q subscales showed no significant associations (all p > 0.05) (table not shown).

Relationship analyses between length of stay (LOS) and EDE-Q

Correlational analyses were conducted to assess associations between length of stay (LOS) and EDE-Q at admission and discharge. The correlations were negative; however, they were not statistically significant. Correlational analyses of length of stay (LOS) and EDE-Q subscales revealed negative associations with shape concern, weight concern, and restraint subscales at both admission and discharge. In contrast, eating concern showed a positive association at admission, which became negative at discharge. The global score was also negative at admission and discharge, and all subscales and the global scores were not statistically significant (table not shown).

Changes in eating psychopathology between admission and discharge

At admission, mean scores on the EDE-Q subscales were as follows: eating concern, 3.97 (SD = 1.24); shape concern, 5.06 (SD = 0.98); weight concern, 4.64 (SD = 1.14); and restraint, 4.21 (SD = 1.90), with a global score of 4.48 (SD = 1.04). The scores at discharge were as follows: eating concern, 1.90 (SD = 1.43); shape concern, 3.67 (SD = 1.76); weight concern, 3.15 (SD = 1.72); restraint, 1.02 (SD = 1.25); and global score, 2.42 (SD = 1.43). All subscales and global scores are significant with p-values of <0.001 (Table 3).

**Table 3:** Mean (±SD) EDE-Q subscale scores at admission and discharge ^a^Significance of comparison according to a paired t-test SD: standard deviation, EDE-Q: Eating Disorder Examination Questionnaire, n: number of participants

	Admission	Discharge	n	p^a^
Restraint	4.21 (±1.90)	1.02 (±1.25)	30	<0.001
Eating concern	3.97 (±1.24)	1.90 (±1.43)	30	<0.001
Shape concern	5.06 (±0.98)	3.67 (±1.76)	30	<0.001
Weight concern	4.64 (±1.14)	3.15 (±1.72)	30	<0.001
Global scores	4.48 (±1.04)	2.42 (±1.43)	30	<0.001

Mean scores on subscales of the EDE-Q by age group are given in Table 4. By following the Fairburn and Cooper method, 30 participants' EDE-Q scores were included for analysis [15]. In this table, the scores on eating concern, weight concern, shape concern, restraint, and the global score are shown to be higher in the 16-24 age group compared to the 25-39 age group at admission being significant at the p < 0.1 level. However, at discharge, the opposite was seen with scores tending to be lower in the 16-24 age group, compared to the 25-39 age group. There were no statistically significant differences between age groups (all p > 0.05). There were significant (p < 0.001) changes in all scores among the age groups between admission and discharge, indicating an improvement in the EDE-Q subscales except for weight concern and shape concern in the 25-39 age group.

**Table 4:** Mean (±SD) scores on the EDE-Q subscales for adolescents and young adults by age group SD: standard deviation, EDE-Q: Eating Disorder Examination Questionnaire, n: number of participants

Age in years	Admission		Discharge	
	16-24 (n = 17)	25-39 (n = 13)	16-24 (n = 17)	25-39 (n = 13)
Restraint	4.39 (±1.55)	3.85 (±2.40)	0.82 (±0.81)	1.35 (±1.73)
Eating concern	4.09 (±1.09)	3.79 (±1.45)	1.54 (±1.23)	2.42 (±1.58)
Weight concern	4.98 (±0.94)	4.26 (±1.30)	2.84 (±1.65)	3.70 (±1.78)
Shape concern	5.38 (±0.67)	4.69 (±1.23)	3.49 (±1.71)	4.10 (±1.79)
Global score	4.71 (±0.67)	4.15 (±1.41)	2.17 (±1.27)	2.89 (±1.57)

## Discussion

This program evaluation assessed a Canadian G-CBT treatment program for two types of EDs, AN and BN, experienced by adolescents and young adult participants. We found that participants showed improvement at discharge after participating in the program. Specifically, as related to eating-disordered behavior (p < 0.001), depression (p < 0.001), anxiety (p < 0.001), and illness cognitions and behaviors (p < 0.001), demonstrating evidence of positive change at a statistically significant level upon completing the program.

The current program evaluation results are consistent with other studies that have demonstrated a strong association between reduced ED symptoms and the use of G-CBT [25,26]. In these prior studies, there was an improvement in restraint, eating concern, and shape concern in adolescents with AN and a significant decrease in all aspects of eating psychopathology in participants with BN at the end of G-CBT treatment. Other research data reported a reduction in ED psychopathology in adolescents and young adults diagnosed with AN [5]. In addition, this particular study used the same G-CBT treatment approach. Similarly, the current program evaluation results support those found by Ohmann et al. in showing that G-CBT is effective in treating adolescent girls with AN [27]. However, contrary to the study of Ohmann et al. [27], this evaluation also showed improvement in shape concern.

Reductions in ED pathology have also been identified as treatment outcomes among those with BN. The study by Jones and Clausen found significant reductions in ED pathology including concerns over body shape, weight, and eating in patients diagnosed with BN [28]. Furthermore, a study by Nevonen and Broberg involving G-CBT and BN participants showed considerable improvements in ED symptoms; however, only small changes were shown for restraint [29]. Along with showing significant reductions in ED pathology, the current program evaluation also revealed substantial improvement in restraint. Consequently, these results provide further support that G-CBT is an effective modality of treatment for EDs, particularly in adolescents.

The current program evaluation findings are also consistent with studies examining ED and mental illness symptoms, specifically, depression, after treatment with G-CBT. In the previous studies, BN and AN participants exhibited improvement in depression and ED symptoms after treatment with G-CBT [27,30,31]. Limited studies have focused on the use of G-CBT targeting anxiety related to EDs [32]. Previous studies have shown a statistically significant relationship between anxiety and disordered eating. In fact, findings from these studies indicate that increases in anxiety also resulted in increases in disordered eating [33,34]. This suggests that a reduction in disordered eating should result in an anxiety reduction, which is consistent with the findings in the current evaluation. One explanation for these reductions in disordered eating and anxiety is that participants acquired coping strategies by the end of the G-CBT treatment. Additionally, the decrease in scores relating to the negative effects of food on the body at discharge also indicated that participants learned to stabilize their eating. In addition to depression and anxiety, trauma and substance use cravings are found to be comorbidities of EDs [35,36]; it is therefore imperative that the program consider undergoing a re-design to include treatment options for these commonly occurring comorbidities.

The program evaluation also found that the clinical significance of restraint, shape concern, weight concern, and eating concern that were identified at admission may no longer be present at discharge. According to Mond et al., a cut-off score of 4 on the global score is generally used as clinically significant [37]. The mean of the global score at admission was above 4, consistent with producing clinical significance, while at discharge, the global score and all the subscales had mean scores that were below 4 at the end of treatment, i.e., the mean of the global score reduced from admission to discharge. These positive findings indicate that at discharge, participants may no longer meet the criteria for an ED. Together, these reported findings demonstrate that the program is very effective in substantially reducing the participants' preoccupation with body image, food, calories, and food avoidance/dietary rules, all of which contribute to EDs. Most importantly, these results offer evidence that coping strategies and eating normalization knowledge can together produce significantly positive outcomes for adolescent participants.

There were several strengths of this program evaluation. First, the evaluation took place in a live treatment environment where individuals receiving G-CBT could be studied in a real-life context. Second, the evaluation used several validated questionnaires commonly used in the field, which added to the validity and reliability of the program evaluation. Third, the evaluation utilized G-CBT, a highly cost-effective treatment modality. Fourth, the evaluation is based on the sole use of secondary data, which has the advantage of lower research costs.

Nonetheless, the evaluation is not without limitations. Some of the data was missing as a result of paper-based data collection. Consequently, the organization transitioned to an electronic data capture system, which should minimize data loss. Also, there were anomalies in some of the responses for questions 13-18 on the EDE-Q. For example, some of the responses were entered as ranges instead of a single number for some of the questions. This resulted in these responses being removed from the evaluation. These exclusions made it impossible to test for differences in the frequency of occurrence of eating-disordered behaviors across age groups. BMI and weight data were also missing from the evaluation. This prevented a conclusion on the effects of the treatment. In addition, the female demographic was the only representation in our sample. It is therefore unclear to what extent these findings may be generalized to males with EDs. There is also a lack of a control group, which limits the ability to attribute these improvements exclusively to G-CBT. Finally, the sample size was small, translating into a lack of good representation for diagnosis type-specific analysis. This was due to the removal of data where some measurement tools were missing entries.

## Conclusions

These findings suggest that group cognitive behavioral therapy (G-CBT) may be an effective intervention for reducing disordered eating behaviors, depression, anxiety, and maladaptive illness cognitions in adolescents and young adults with eating disorders, depression, and anxiety. Further research is necessary using data from the EDE-Q to determine whether the results remain consistent and compare the intervention effects across age groups. Moreover, a follow-up study is needed to investigate the extent to which participants maintained their recovery. Controlled studies are also needed to better isolate the effects of G-CBT to check for connections between intervention and observed outcomes. Finally, more broad measures need to be incorporated into future assessments of clinical outcomes in programs like these to ensure that they include all relevant issues, particularly those associated with serious negative outcomes. The overall results from this evaluation indicate that to achieve recovery from AN or BN in adolescents and young adults, interventions are needed that target both behavioral and psychological issues.
